# Editorial on Special Issue “Advances and Novel Treatment Options in Metastatic Melanoma”

**DOI:** 10.3390/cancers14030707

**Published:** 2022-01-29

**Authors:** Alessandra Carè, Donatella Del Bufalo, Antonio Facchiano

**Affiliations:** 1 Center for Gender-Specific Medicine, Istituto Superiore di Sanità, Viale Regina Elena 299, 00161 Rome, Italy; alessandra.care@iss.it; 2 Preclinical Models and New Therapeutic Agents Unit, IRCCS Regina Elena National Cancer Institute, Via Elio Chianesi 53, 00144 Rome, Italy; donatella.delbufalo@ifo.it; 3 Laboratory of Molecular Oncology, Istituto Dermopatico dell’Immacolata, IDI-IRCCS, Via Monti di Creta 104, 00167 Rome, Italy

Investigating mechanisms controlling melanoma setup, development and progression is currently an extremely hot and rapidly evolving topic. Immunotherapy- and target therapy-based approaches are expected to substantially improve the survival in melanoma, as well as several other cancer types, changing the prognosis in many cancer patients. This is fostering a large interest in the detailed analysis of melanoma underlying mechanisms, to support the identification of more effective markers and therapeutic approaches. The Special Issue published in 2020–2021 by Cancers, entitled “Advances and Novel Treatment Options in Metastatic Melanoma”, collects 24 manuscripts from Authors working in several different Institutions acting in different nations. According to the official page (https://www.mdpi.com/journal/cancers/special_issues/metastatic_melanoma, accessed on 18 January 2022), the Special Issue raised a very high interest in readers, and the number of views and citations of each manuscript is constantly increasing. The Special Issue addresses, under different aspects, the biology of melanoma and the way it affects its onset, development, and response to therapy. Manuscripts can be categorized under three main fields, namely: (1) mechanisms underlying melanoma setup and development, (2) melanoma therapeutic approaches, and (3) melanoma biomarkers.

An overview of each manuscript is reported below.

## 1. Mechanism Underlying Melanoma Setup and Development

Recently, there have been discoveries of new factors involved in melanoma progression and response to therapy, and their potential as pharmacologic targets. Among these, the myristoylated alanine-rich c-kinase substrate, MARCKS, which in addition to its role as an indicator of the protein kinase C activity, also plays a pivotal role as regulator of the metastatic behaviour of melanoma through its activation by WNT5A [1]. Also, BCL2L10, an antiapoptotic protein belonging to the bcl-2 family, has been identified both in melanoma cell lines and tumor samples, and its role as a pro-survival factor in melanoma has been reported [2]. Blockade of carbonic anhydrases XII (CAXII) under low oxygen condition induced a negative effect on melanoma cell migration and invasion, paralleled by a reduction of FAK phosphorylation and metalloprotease activities, thus suggesting CAXII as a possible therapeutic target for melanoma [3].

Melanoma pathobiology and response to target therapy or immunotherapy strictly depends on cells present in the tumor microenvironment (TME), such as fibroblasts, endothelial and immune cells [4]. A dynamic crosstalk between tumor cells and stroma plays a fundamental role in melanoma initiation, progression and metastasis [5]. TME affects melanoma progression also through the presence of basic or acidic extracellular matrix components released by melanoma or stromal cells. To this regard, in metastatic melanoma patients, plasmacytoid dendritic cells (pDCs), which play a relevant function in the anti-tumor immune response, are strongly impaired in terms of viability and function by lactic acidosis. BRAF (BRAFi) and MEK (MEKi) inhibitors partially rescued pDCs function in melanoma patients carrying BRAFV600 mutations [6].

Even if several mechanisms of intrinsic and acquired resistance to melanoma treatment have been identified, resistance still remains a threat for melanoma patient’s survival [7]. Cancer stem cells (CSCs) represent one of the causes for development of resistance, thus contributing to the disease relapse following an initial response. A bioinformatic approach led to the identification of four major clusters of CSCs in BRAF mutated melanoma [8].

## 2. Melanoma Therapeutic Approaches

Treatment of metastatic melanoma still represents a challenge due to rapid dissemination and drug resistance. In the last decade, many novel therapeutic options have been developed and others are ongoing with important improved outcomes for patients. Major results are based on target- and immuno-therapies, including the option of novel combination treatments. 

In the era of precision medicine, the first important novelty was represented by BRAF inhibitors acting on BRAF-mutated melanoma cells to selectively inhibit the MAPK pathway. More recent data evidenced the relevance of the immunomodulatory effects of BRAFi, including the capability to increase levels of immunostimulatory cytokines and decrease the immunosuppressive ones, enhance melanoma differentiation and presentation of tumor antigens, paralleled by increased intra-tumoral T-cell infiltration and activity [9]. Looking at adverse events, evaluated in the BRAFi plus MEKi combination, beside the most common pyrexia, fatigue, nausea, rash and hypertension, patients showed arthralgia. More than 30% of the treated patients reported arthralgia mostly affecting small joints and resembling symptoms associated with rheumatoid arthritis. It is interesting to note that occurrence of arthralgia was often associated with a better prognosis [10]. Going further in the development of additional strategies, promising approaches are based on the inhibition of protein tyrosine phosphatases, also in view of the effects on tumor infiltrates [11], and on the molecular targeting of the human antigen R (HuR), which stabilizes several oncoproteins. Interestingly, HuR inhibition in melanoma exhibited antitumor activity independently from the BRAF mutational status [12]. 

Based on recent progress in nanotechnology, nanoparticles represent another important therapeutic approach now moving to therapy. Indeed, nanovesicles can be loaded with a therapeutic cargo and modified in order to improve their entry and accumulation into the tumor, in turn preventing their content from degradation [13]. In addition, novel approaches based on so called “smart nanoparticles” are now under clinical trials in melanoma patients. These nanoparticles display higher affinity for melanoma components reducing toxic effects and aspecific targeting [14]. A growing interest is now based on natural products as antineoplastic drugs. Among them, promising results are showing the effectiveness of essential oil as complementary or alternative drugs for several diseases and as factors improving quality of life of cancer patients [15].

More complex is treatment of advanced mucosal and uveal melanomas still lacking effective therapeutic options. In advanced mucosal melanoma, immunotherapy and radiotherapy results, based on retrospective studies, showed the efficacy of anti-PD1 antibodies and the beneficial effects of combination with radiotherapy. Even so, median overall survival was 16.3 months [16]. Seemingly, the situation for uveal melanoma shows survival rates of approximately one year for nearly 50% of patients [17].

## 3. Melanoma Biomarkers

In the Special Issue several studies address the identification of markers able to effectively characterize melanoma patients. In the study published by Cesati et al. [18], the protein and gene expression of 27 cytokines, has been evaluated in the serum and in the tissue biopsies, respectively, of hundreds of patients and healthy controls. Different statistical approaches were applied leading to the identification of molecules in the serum able to significantly characterize patients vs. controls, as well as patients as function of gender and Breslow thickness. A striking feature is reported at the gene expression level. In fact, this study identifies, for the first time, a 4-gene signature able to discriminate patients form controls, with an extremely high accuracy (AUC = 0.98). A study by Aladowicz et al. [19] investigated metastatic melanoma, addressing metastasis-specific traits and metastasis-specific signaling pathways. From a patient derived xenografts collection, authors study the role of the adaptor protein ShcD, reporting its role in controlling melanoma spreading and its adhesion to the extracellular matrix. Overexpression of this factor has been found to increase melanoma cells movement via RAC1 signaling and DOCK4 confinement in the cytoplasm. Therefore, ShcD expression values are proposed to be investigated to predict the metastatic potential of melanoma cells. Metastatic features of melanoma cells were also related to the expression of the two-pore channel 2 (TPC2) [20]. The study shows that TPC2 knockout enhances metastatic potential of the melanoma cells consistently to increased expression of ZB-1, Vimentin, N-Cadherin and MMP9, and indicates the involvement of ORAI1/Ca^2+^/PKC-βII pathway. The study shows that TPC2 reduction observed in metastatic patients opens new diagnostic hypotheses in metastatic patients. The manuscript by Pilla et al. [21] reviews the current status of investigation on biomarkers useful for melanoma patients stratification and to predict patients’ responsiveness to therapy and other clinical outcomes. Biomarkers discussed belong to the classical melanoma-related markers, as well as to immune factors, microbiome-related molecules, mutations status, microenvironment-derived molecules, circulating DNA and others. The manuscript also discusses how biomarkers investigation may help the understanding of molecular basis of melanoma and the identification of new therapeutic approaches. The review by Lucianò and Tata [22] summarizes and points out recent findings regarding the role of cholinergic receptors outside the nervous system, namely in controlling the proliferation, apoptosis, angiogenesis, and epithelial mesenchymal transition in different cancer types including melanoma. The role of such receptors is also discussed in controlling metastatic features of melanoma. The review by Sacco et al. [23] highlights the role of circulating DNA in both early melanoma diagnosis and in the clinical management of more advanced phases. In fact, evaluation of circulating DNA can monitor aggressiveness of the resected melanoma, as well as minimal residual disease, recurrence, clinical progression, response and resistance to therapy. The importance of biomarkers such circulating DNA is also highlighted for patients stratification purposes. Finally, the review by Bellenghi et al. [24] underlines sex- and gender-related diversities in melanoma patients. The manuscript also highlights different incidence, anatomic localization, expression of sex-hormones as well as different expression of immune related genes located on the X chromosome. Additional evidences are discussed since different response to immunotherapy are reported, showing better improvements in men than in women. Such data highlight the possible consequences of unbalanced recruitment in clinical studies addressing the efficacy of new immunotherapy approaches.
